# Data related to the experimental design for powder bed binder jetting additive manufacturing of silicone

**DOI:** 10.1016/j.dib.2018.04.068

**Published:** 2018-04-23

**Authors:** Farzad Liravi, Mihaela Vlasea

**Affiliations:** Multi-Scale Additive Manufacturing Laboratory, Department of Mechanical and Mechatronics Engineering, University of Waterloo, Waterloo, ON, Canada N2L 3G1

## Abstract

The data included in this article provides additional supporting information on our recent publication (Liravi et al., 2018 [1]) on a novel hybrid additive manufacturing (AM) method for fabrication of three-dimensional (3D) structures from silicone powder. A design of experiments (DoE) study has been carried out to optimize the geometrical fidelity of AM-made parts. This manuscript includes the details of a multi-level factorial DOE and the response optimization results. The variation in the temperature of powder-bed when exposed to heat is plotted as well. Furthermore, the effect of blending ratio of two parts of silicone binder on its curing speed was investigated by conducting DSC tests on a silicone binder with 100:2 precursor to curing agent ratio. The hardness of parts fabricated with non-optimum printing conditions are included and compared.

**Specifications table**

**Table:** 

Subject area	Engineering, Materials Science
More specific subject area	Additive Manufacturing
Type of data	Table, figure
How data was acquired	Design of Experiments, Thermocouple
Data format	Raw, Analyzed
Experimental factors	The samples were 3D printed based on the experimental design factor treatments in a completely randomized fashion.
Experimental features	For geometrical fidelity optimization, the effects of different values of two factors (layer thickness (LT) and binder dispensing frequency (Fr)) on height and diameter of 3D printed cylinders were studied. The effects of factors on all three responses were simultaneously investigated using desirability function method.
	For measurement of powder-bed’s temperature a thermocouple was used.
	The crosslinking kinetics of 100:2 silicone binder was studied using a DSC at isothermal temperatures of 85, 90, 95, and 100 ^o^C.
	A handheld durometer was used for Shore 00 hardness tests.
Data source location	Multi-Scale Additive Manufacturing Laboratory, University of Waterloo, Waterloo, ON, Canada.
Data accessibility	This article.
Related research article	Liravi et al., 2018 [1]

**Value of the Data**

• The raw data of dimensional features provided in Table 1 provides the readers with the chance of fact checking the results by following the analysis steps.

**Table 1:** The measured values for the H, ID, and DD for the experimental design.

Standard Order	Run Order	LT	Fr	*H* (μm)	ID (μm)	DD (μm)
9	1	50	300	4130.676	5407.209	1563.818
1	2	50	100	5685.574	6966.943	1476.194
7	3	50	100	5907.289	6904.469	1256.984
12	4	100	300	3673.833	5579.145	1283.139
6	5	100	300	3863.966	5329.552	1930.681
8	6	50	200	3852.614	5685.995	1905.692
4	7	100	100	4894.481	7436.540	2126.095
3	8	50	300	4116.611	6160.803	2074.107
2	9	50	200	3909.257	6762.391	1615.955
11	10	100	200	3619.815	6588.914	1835.162
5	11	100	200	3645.568	6307.557	1137.825
10	12	100	100	5904.600	7353.762	1869.109
• The desirability function response optimization (Table 5) shows the values of LT and Fr (in the investigated region) resulting in dimensional features closest to their target values.
• The temperature vs. time data provided in Fig. 1 supports our interpretation of thermal analysis of silicone binder using differential calorimetry scanning (DSC).

**Fig. 1:**
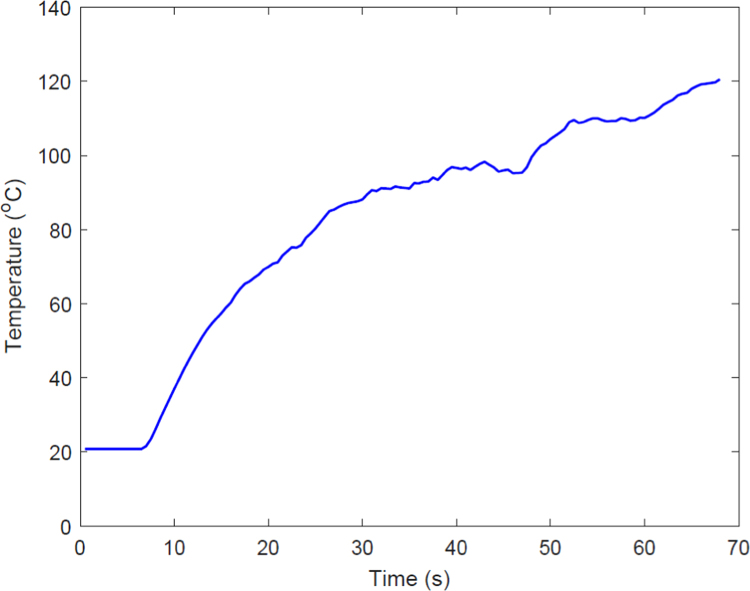
Temperature of the powder bed vs. time under heat lamp exposure.
• The thermal behavior of 100:2 silicone binder provided in Fig. 2 shows that increasing the amount of curing agent does not speed up the full crosslinking process, however, it reduces the crosslinking initiation temperature.

**Fig. 2:**
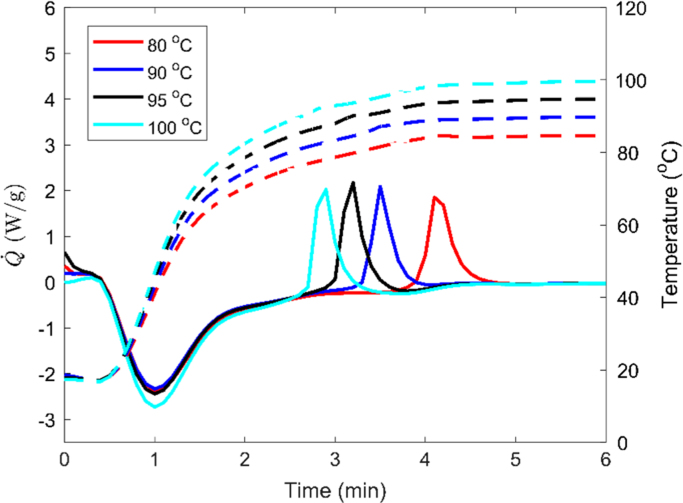
Thermal analysis results for silicone binder with 100:2 precursor to curing agent ratio.
• The comparison of hardness values shown in Fig. 3 and Table 6, Table 7, Table 8, Table 9, Table 10 is indicative of the insignificant effect of process parameters on the hardness of fabricated parts for the selected silicone binder and powder.

**Fig. 3:**
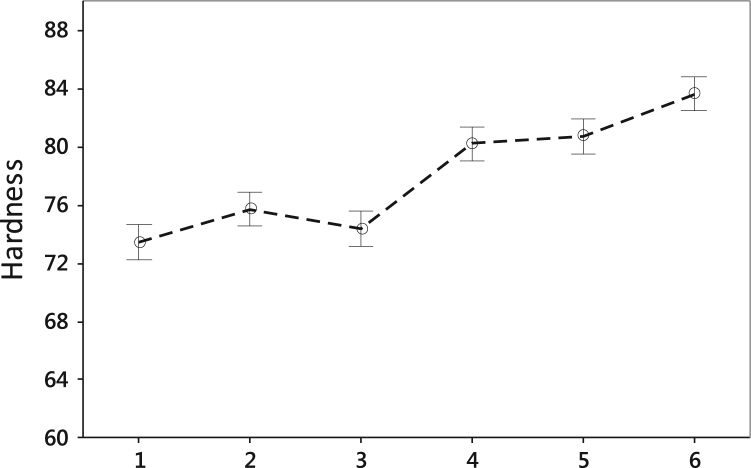
Comparing the average and standard deviation of hardness measurements for: (1) 50 μm and 1 drop per 100 μm; (2) 50 μm and 1 drop per 200 μm; (3) 50 μm and 1 drop per 300 μm; (4) 100 μm and 1 drop per 100 μm; (5) 100 μm and 1 drop per 200 μm; and (6) 100 μm and 1 drop per 300 μm.

## 1 Data

In order to optimize the 3D printing parameters, a multi-level experimental design was formed with layer thickness (LT) and dispensing frequency (Fr) of the silicone binder deposition as the control factors. The height (*H*), inner diameter (ID), and the diameter difference (DD) between the inner and outer circles fitted to the cross section of parts are the responses. The outer diameter (OD) is the diameter of the largest circle fitted to the cross-section of the cylindrical parts so that it covers the entire cross-section including the irregular edges. The diameter of the circle that only covers the central parts of the cross-section and not the irregularity caused by the lateral infiltration of silicone binder is ID. The structure of DoE and the measurement details are provided in Table 1. The analysis of variance (ANOVA) results are shown in Table 2, Table 3, Table 4 for H, ID, and DD, respectively.

**Table 2:** ANOVA results for the average height.

Source	Degree of Freedom	Adjusted Sum of Squares	Adjusted Mean Square		*F*-Value	*P*-Value
Model	5	8550286	1710057	18.49		0.001
Linear	3	8538548	2846183	30.78		0
LT	1	333253	333253	3.6		0.106
Fr	2	8205295	4102648	44.36		0
2-Way Interaction	2	11738	5869	0.06		0.939
LT × Fr	2	11738	5869	0.06		0.939
Error	6	554859	92476			
Total	11	9105145

**Table 3:** ANOVA results for the inner diameter.

Source	Degree of Freedom	Adjusted Sum of Squares	Adjusted Mean Square	*F*-Value	*P*-Value
Model	5	5160132	1032026	6.59	0.02
Linear	3	4831906	1610635	10.29	0.009
LT	1	41732	41732	0.27	0.624
Fr	2	4790174	2395087	15.3	0.004
2-Way Interaction	2	328226	164113	1.05	0.407
LT×Fr	2	328226	164113	1.05	0.407
Error	6	939373	156562		
Total	11	6099505

**Table 4:** ANOVA results for the diameter differences.

Source	Degree of Freedom	Adjusted Sum of Squares	Adjusted Mean Square	*F*-Value	*P*-Value
Model	5	534849	106970	0.94	0.516
Linear	3	23421	7807	0.07	0.975
LT	1	6973	6973	0.06	0.813
Fr	2	16449	8224	0.07	0.931
2-Way Interaction	2	511428	255714	2.25	0.187
LT × Fr	2	511428	255714	2.25	0.187
Error	6	682013	113669		
Total	11	1216862			

The path to the optimized region for each parameter was found using the response surface method. Finally, all three responses were optimized simultaneously using desirability function technique (utility transfer function). The optimization results are demonstrated in Table 5. The levels of significant factors were selected so that DD was minimized, and *H* and ID approached the target values of 3 mm and 5 mm, respectively.

**Table 5:** Desirability function response optimization.

Response	Goal	Lower	Target	Upper	Weight
DD	Minimμm	*	1137.82	2126.09	1
ID	Target	4500	5000	7436.54	1
*H*	Target	2700	3000	5907.29	1

The DSC results for the silicone binder reveal that it gets cured almost immediately at a temperature in the range of 100–110 °C. In order to make sure this polymerization temperature is reached in 60 s, the temperature of powder bed was measured using a thermocouple. The temperature increase is plotted in Fig. 1 .

**Table 6:** The durometry results for the 3D printed cylinders. Printing condition: 50 μm layer thickness and 1 drop per 100 μm dispensing frequency (*n* = 3).

Sample	Hardness (shore 00) 50 μm | 1 drop per100 μm			
	Test 1	Test 2	Test 3	Average
Cylinder 1 (batch 1)	72.4	72.1	79.8	74.77
Cylinder 2 (batch 1)	68.5	70.2	70	69.57
Cylinder 3 (batch 1)	69.5	75.1	74.6	73.07
Cylinder 1 (batch 2)	76.1	75.1	75.5	75.57
Cylinder 2 (batch 2)	74.3	72.2	75.1	73.87
Cylinder 3 (batch 2)	70.7	77.8	73.2	73.90
Total average for cylindrical samples				**73.46**

**Table 7:** The durometry results for the 3D printed cylinders. Printing condition: 50 μm layer thickness and 1 drop per 200 μm dispensing frequency (*n* = 3).

Sample	Hardness (shore 00) 50 μm | 1 drop per 200 μm			
	Test 1	Test 2	Test 3	Average
Cylinder 1 (batch 1)	75.2	75.7	78.8	76.57
Cylinder 2 (batch 1)	76.2	73.7	70.1	73.33
Cylinder 3 (batch 1)	75.9	76.8	75.5	76.07
Cylinder 1 (batch 2)	73.4	77.1	76.1	75.53
Cylinder 2 (batch 2)	76.8	76.1	75.8	76.23
Cylinder 3 (batch 2)	75.4	77.9	76.8	76.70
Total average for cylindrical samples				**75.74**

**Table 8:** The durometry results for the 3D printed cylinders. Printing condition: 50 μm layer thickness and 1 drop per 300 μm dispensing frequency (*n* = 3).

Sample	Hardness (shore 00) 50 μm | 1 drop per 300 μm			
	Test 1	Test 2	Test 3	Average
Cylinder 1 (batch 1)	77.2	72.3	72.9	74.13
Cylinder 2 (batch 1)	73.3	73.4	74.9	73.87
Cylinder 3 (batch 1)	78.5	71.3	74.6	74.80
Cylinder 1 (batch 2)	71.3	77.5	71.1	73.30
Cylinder 2 (batch 2)	76.6	79.9	78.9	78.47
Cylinder 3 (batch 2)	72.1	72.5	70.7	71.77
Total average for cylindrical samples				**74.39**

**Table 9:** The durometry results for the 3D printed cylinders. Printing condition: 100 μm layer thickness and 1 drop per 100 μm dispensing frequency (*n* = 3).

Sample	Hardness (shore 00) 100 μm | 1 drop per 100 μm			
	Test 1	Test 2	Test 3	Average
Cylinder 1 (batch 1)	80.9	80.6	78	79.83
Cylinder 2 (batch 1)	80	76.1	80.1	78.73
Cylinder 3 (batch 1)	85.8	78.5	76.1	80.13
Cylinder 1 (batch 2)	81.9	87	79.6	82.83
Cylinder 2 (batch 2)	77.9	76.9	88.6	81.13
Cylinder 3 (batch 2)	80.5	79.4	76.4	78.77
Total average for cylindrical samples				**80.24**

**Table 10:** The durometry results for the 3D printed cylinders. Printing condition: 100 μm layer thickness and 1 drop per 200 μm dispensing frequency (*n* = 3).

Sample	Hardness (shore 00) 100 μm | 1 drop per 200 μm			
	Test 1	Test 2	Test 3	Average
Cylinder 1 (batch 1)	76.4	84.5	76	78.97
Cylinder 2 (batch 1)	82.8	82.2	76.5	80.50
Cylinder 3 (batch 1)	79.3	79.5	84.1	80.97
Cylinder 1 (batch 2)	83.5	82.2	78.6	81.43
Cylinder 2 (batch 2)	81.6	83.6	81.6	82.27
Cylinder 3 (batch 2)	81.7	76.3	83.1	80.37
Total average for cylindrical samples				**80.75**

## 2 Experimental design, materials, and methods

To measure the temperature of powder-bed, a thermocouple was fixed on the surface of the feeding chamber filled with silicone powder using a Kapton tape. The powder-bed temperature was increased by exposing it to the heat provided by a thermal lamp. The temperature values were transferred to a computer using a data acquisition device (NI USB-6009, National Instrμments, TX, USA), and recorded using an in-house developed program in LabView environment.
