# Supplementary material for: Data related to the experimental design for powder bed binder jetting additive manufacturing of silicone
Source: Data Brief. 2018 Apr 23;18:1477–83. doi: 10.1016/j.dib.2018.04.068 (PMC5998170; doi:10.1016/j.dib.2018.04.068)
Supplement: Supplementary file 1 — Supplementary material [file mmc1.docx]

Dear Editors,

There were no available conflict of interest forms to be submitted for the Data in Brief.

The authors would like to declare that there are no additional conflicts of interest associated with this Data in Brief outside of the ones declared for the original journal submission associated with the Journal Paper for this Data in Brief.

To avoid the error message on the submission page, we have submitted this form.

Best regards,

The Authors
